# Exploring the role of agentic AI in fostering self-efficacy, autonomy support, and self-learning motivation in higher education

**DOI:** 10.3389/frai.2026.1738774

**Published:** 2026-01-22

**Authors:** Jehad Alqurni

**Affiliations:** Department of Educational Technologies, College of Education, Imam Abdulrahman Bin Faisal University, Dammam, Saudi Arabia

**Keywords:** agentic AI, artificial intelligence, autonomy support, higher education, self-efficacy, self-learning motivation

## Abstract

**Introduction:**

Rapid adoption of Artificial Intelligence (AI) in learning has revolutionized learners’ engagement but comprehension of psychological and technological drivers of successful AI-enabled learning remains scarce. This research investigates how students’ perceived agency of AI, usefulness, ease of use, trust, autonomy supporting, and self-efficacy collectively impact students’ self-learning behavior and motivation. Based on Technology Acceptance Model (TAM), Social Cognitive Theory (SCT), and Self-Determination Theory (SDT) theories, our research model predicts an integrated model of motivational and behavioral processes underlying AI adoption in learning settings.

**Methods:**

We adopted and followed a quantitative research design with a structured questionnaire administered among 280 higher education students in Saudi Arabia. We applied Structural Equation Modeling (SEM) using SmartPLS 4 to analyze data.

**Results:**

Findings indicate that students’ perceived agency of AI significantly predicts usefulness, ease of use, and autonomy supporting, while ease of use significantly enhances AI-enabled self-efficacy. Self-efficacy and autonomy supporting significantly impact self-learning motivation, driving self-learning behavior positively. But usefulness and trust in AI failed to influence self-efficacy directly, which reveals cultural and contextual settings.

**Discussion:**

This research adds richness to the fusion of TAM, SCT, and SDT theories in illustrating how AI’s perceived autonomy and usability collectively promote self-directed learning motivation. This research also provides guidelines to educators and system designers to design AI tools that promote learner autonomous settings, usability, and confidence. Future research ought to perform longitudinal and cross-cultural validations to fine-tune theoretically.

## 1 Introduction

Over the past few decades, education has changed significantly due to the influence of computer and communication technologies. Traditional classrooms, characterized by teacher-centrated learning, static and fixed curriculum, same-paced learning, and minimal feedback, have failed repeatedly to accommodate differentiated students (Dahri et al., 2024; Almogren et al., 2024a). Students have remained disengaged, unable to influence the direction of learning, and restricted by narrow assessment procedures. Traditional systems generally make assumptions that all students learn at equal rates, profit from the same teaching methodology, and need equal facilitation by instructors (Dahri et al., 2024; Almogren et al., 2024a). This homogeneity ignores individual variability in background knowledge, motivation, self-regulation ability, learning modes, and learning rate. Furthermore, feedback in most traditional contexts comes late, generic, and inadequate to students’ constantly changing needs (Kalantzis and Cope, 2004). All these have been increasingly challenged in research on education, due to the inhibitions that they create on students’ motivation and learning of self-regulated behaviors, which are critically needed in higher education and in life continuing learning contexts (de Bruijn-Smolrs et al., 2016; Roth et al., 2016).

With the rise in digital instrumentation, Internet learning platforms, intelligent tutoring systems, adaptive learning systems, and AI-driven learning technologies, Strielkowski et al. (2025) noted that there is growing enthusiasm about how technology could make up some of those weaknesses. Technology innovations such as Learning Management Systems (LMS), MOOCs, interactive multimedia, and simulation-based learning have begun to allow greater flexibility in pacing, diversity in modality in instructions, immediate feedback, and access to more kinds of resources (Munna et al., 2024; Ok, 2025). Studies have found that where digital technology is well crafted, it has been demonstrated to enhance students’ engagement, cognitive learning outcomes, and satisfaction. But many of those tools still function in reactive or prescriptive modes: students respond to system directions, yet moderate adaptation on the part of the system is small, underpinned all too often by only simple heuristics or pre-defined branching logic. Such tools have the potential to increase utility and ease of access, but do not necessarily encourage deeper motivational constructs—such as perceived agency, self-determination or self-efficacy—and may little encourage true self-directional learning behavior.

Recent advances in Artificial Intelligence (AI), notably in agentic AI, adaptive systems, reinforcement learning, large-language models, and explainable AI, have opened new possibilities in educational settings. Agentic AI refers to AI systems that can act with a degree of autonomy: making decisions, adapting dynamically to learner needs, guiding learning paths proactively, offering personalized scaffolding, and even initiating interventions or suggestions rather than merely responding to users. In education, these systems include adaptive tutoring platforms, AI agents that monitor student progress and provide timely feedback, and intelligent companions capable of supporting students’ decision making about what, when, and how to learn. Educational research has begun to document benefits of AI for increasing self-efficacy and motivation. For example, a systematic review found that AI tools significantly contribute to the development of computational thinking and self-efficacy among learners across levels when the systems adapt to learner performance and provide supportive feedback (Massaty et al., 2024). Another study of nursing students in China showed that AI literacy correlated positively with AI self-efficacy, which in turn was linked to higher engagement (He et al., 2025). Similarly, investigations among pre-service special education teachers in China have revealed that perceived usefulness and ease of use influence their intention to adopt AI tools, mediated by self-efficacy (Yao and Wang, 2024). Moreover, teacher studies in K-12 contexts indicate that while attitudes toward AI are generally positive, actual readiness, measured via self-efficacy, access, and support—varies widely across individuals and institutional contexts (Bergdahl and Sjöberg, 2025). These findings suggest that agentic AI holds promise not only for content delivery but for motivational and self-regulatory dimensions of learning. Prior studies have predominantly focused on China, Europe, and Western contexts, with limited empirical evidence from Middle Eastern higher education systems (Dahri et al., 2025; Dahri et al., 2025; Bandi et al., 2025).

Despite these promising developments, there remains a lack of clarity around exactly *how* different perceptions of an AI system (such as its perceived agency, usefulness, or ease of use), together with factors like trust, autonomy support, and self-efficacy, combine to influence students’ self-learning motivation and ultimate self-learning behavior. While prior work has examined pairwise relationships (for example, AI literacy → engagement, or usefulness → behavioral intention), comprehensive models that integrate these constructs and test mediated pathways are relatively rare (Fan and Zhang, 2024; Wang et al., 2025; Gkanatsiou et al., 2025; Han et al., 2025). Furthermore, many studies are localized to particular domains, such as language learning, special education, or higher vocational education, leaving out broader student populations and contexts (Tyler et al., 2004; Gersten and Woodward, 1994; Boud and Walker, 1998). Also, there is little empirical work on the role of perceived agency of AI—that is, how much students view the AI tools as acting independently or adaptively—and how that perceived agency interacts with trust, autonomy support, and self-efficacy to drive motivation and behavior. Theoretical perspectives from Social Cognitive Theory (which emphasizes self-efficacy, observational and mastery experience) (Schunk, 2012; Stajkovic and Luthans, 1998) and Self-Determination Theory (which emphasizes autonomy, competence, and relatedness) offer useful lenses for this investigation (Rigby and Ryan, 2018; Moore et al., 2020; Vansteenkiste et al., 2018). Integrating these theories in a model that includes agentic AI notions promises to yield richer understanding of motivational and behavioral dynamics in AI-supported self-learning. To address these gaps, the present study proposes and empirically validates a comprehensive structural model that integrates perceived AI agency, autonomy support, trust, and AI-supported self-efficacy to explain students’ self-learning motivation and self-learning behavior in AI-assisted educational settings. By doing so, this study extends existing AI adoption research beyond intention-based models and offers context-specific empirical insights into how agentic AI tools shape meaningful learning behaviors. In particular:

1. To define the associations between perceived agency of AI and (a) perceived usefulness, (b) ease of use, and (c) autonomy support.
2. To understand how perceived usefulness, ease of use, and trust in AI act to construct AI-assisted self-efficacy.
3. To examine how self-efficacy and autonomous support enhance self-learning motivation.
4. A systematic review of how self-learning motivation affects self-learning behavior in AI-assisted learning settings.
5. To test mediated associations between these constructs, pinpointing indirect associations between perceptions of AI and true self-learning behavior.

According to these aims, the research answers the following research questions:

- **RQ1:** How do students’ conceptions of AI’s agency affect students’ conceptions of usefulness, ease of use, and facilitation of autonomy?
- **RQ2:** How do perceptions of usefulness, ease of use, and trust in AI influence students’ self-efficacy with AI?
- **RQ3:** In what ways do students self-learning and self-efficacy benefit from autonomy
- **RQ4:** How does self-learning motivation influence real self-learning behavior through motivational and perceptual intervening constructs?
- **RQ5:** What indirect channels (mediations) play important roles in connecting perceptions regarding AI to learning behavior of learners?

This research contributes to theory, practice, and policy. From a theoretical standpoint, it advances knowledge on agentic AI by placing measures of perceived agency, trust, and autonomous motivation under a Structural Equation Modeling (SEM) framework based on Social Cognitive Theory and Self-Determination Theory. From an empirical point of view, it gathers data from diverse higher education students to provide understanding on how perceptions get translated into motivation and self-learning in AI-enacted contexts. From a practical point of view, findings shall assist learning AI systems designers to identify strong traits, such as augmenting perceived agency, usability, autonomy, and trust—these strengthen self-learning. Finally, policy decisions shall guide institutions and planners in establishing standards, allotment of funds, and designing professional development events to ensure that AI tools advance learning motivation and autonomy and not dependency or superficiality.

## 2 Theoretical background and literature review

This study draws primarily on three theories: Technology Acceptance Model (TAM), Social Cognitive Theory (SCT), and Self-Determination Theory (SDT). The TAM (Davis, 1989) posits that two core beliefs, perceived usefulness (PU) and perceived ease of use (PEU), are key determinants of users’ attitudes toward adopting and using technology, which then lead to behavioural intention and actual use (Tam, 2024; Dahri et al., 2024; Mun et al., 2006; Pan and Jordan-Marsh, 2010) defined PU as the degree to which an individual believes that using a specific technology enhances their performance, while PEU is the degree to which using that technology is free of effort (Yen et al., 2010). TAM has been widely applied in educational technology studies to explain students’ and teachers’ adoption of e-learning, AI tools, and ICT more broadly. For example, pre-service teachers’ intention to adopt generative AI has been modelled via extended TAM showing strong paths from PU and PEU to behavioral intention (Şimşek et al., 2025; Falebita and Kok, 2025). Social Cognitive Theory (Bandura, 1986) emphasizes that learning occurs in a social context with dynamic and reciprocal interaction of person, environment, and behavior. Key in SCT is self-efficacy, the belief in one’s capabilities to organize and execute the courses of action required to manage prospective situations. In education, self-efficacy has been shown to influence motivation, persistence, strategy use, and ultimately learning outcomes. SCT also supports consideration of how trust and agency (agency meaning control, autonomy, or action) influence beliefs and behaviors. Whereas Self-Determination Theory (Deci and Ryan, 2000) focuses on psychological needs: autonomy, competence, and relatedness. When these needs are satisfied, intrinsic motivation and engagement are higher. In technology-enhanced learning settings, SDT has been used to explore how autonomy support (from tools or instructors) and competence (often via self-efficacy) foster students’ motivation and self-regulated learning. Together, these theories provide a strong foundation for analyzing how perceptions of an AI system (agentic or autonomous AI) combine with beliefs and environmental/psychological supports to predict motivation and behavior. Below is a summary of selected existing studies that examine constructs similar to those in this model (PU, PEU, self-efficacy, autonomy, trust, motivation, behavior with AI or technology in education) (Table 1).

**Table 1:** Summary of prior empirical studies Related to technology acceptance.

Study	Context / Sample	Key variables examined	Main findings relevant to our model
Falebita and Kok (2025)	Undergraduates in STEM / higher ed	PEU, PU, self-efficacy, attitudes toward AI tools	PEU significantly influences PU; both PEU and PU predict attitudes and readiness; self-efficacy mediates or moderates adoption.
Şimşek et al. (2025)	Pre-service teachers	PU, PEU, Learning Motivation, metacognitive self-regulation	PU and PEU strong predictors of intention; Learning Motivation mediates relationships; PEU influences PU significantly.
Chen et al. (2025)	Undergrad students in China	Novelty-seeking, self-efficacy, PU, PEU, attitude to robots	Self-efficacy enhances PEU; PU and PEU predict attitude and intention toward a novel AI technology.
Zhang et al. (2023)	Pre-service teachers	PU, PEU, intention to use AI	PU and PEU significantly affect intention; PU has stronger effect.
Xia et al. (2023)	Grade 9 students in K-12	Autonomy, competence (psychological needs), AI knowledge	Autonomy and competence needs support SRL with AI; effects moderated by gender and AI knowledge.
Khan et al. (2024)	Public sector / operators	Trust in AI, AI self-efficacy, intent to adopt decisions	Trust influences self-efficacy which in turn predicts behavioral intention; importance of trust and efficacy.
Tuffahati and Nugraha (2021)	Students using Google Classroom	PU, PEU, learning motivation	Both PU and PEU have significant positive effects on learning motivation; PU tends to have stronger effect.
Massaty et al. (2024)	Students across levels and disciplines	AI tools, computational thinking, self-efficacy	AI tools help boost self-efficacy; stronger effects when tools adapt to learner and provide scaffolding; implications for designing AI to support competence.

These studies collectively indicate that PU and PEU are robust predictors of attitudes/intentions/usage in educational contexts involving AI or other technologies; that self-efficacy is a critical mediator, especially under SCT; that autonomy and psychological need satisfaction (from SDT) matter for motivation and self-regulated/self-directed learning; and that trust (in AI or technology) is increasingly being included, showing importance. Given the precedents, this study includes the following key latent variables, each of which has theoretical and empirical rationale:

- Perceived Agency of AI: This captures how much students believe the AI tool acts adaptively, initiatively, or independently. While fewer studies have directly measured agencies, related notions of autonomy support, control, or adaptivity are emerging (Hidayat-ur-Rehman, 2024). Agentic properties may enhance users perceived usefulness, self-efficacy, and autonomy, aligning with both SCT and SDT.
- Perceived Usefulness (PU): Central in TAM, PU reflects beliefs about performance enhancement (Pan and Jordan-Marsh, 2010; Yen et al., 2010). In education, believing a tool will help in academic performance strongly influences motivation and uptake. Seen in many studies above (e.g., generative AI in teacher studies; Google Classroom; technology readiness). High PU likely boosts self-efficacy (believing the tool will help me succeed), autonomy perception, and motivation.
- Perceived Ease of Use (PEU): Also central in TAM, PEU influences PU and reduces barriers. When a tool is easy to use, students can focus on learning rather than struggling with interface or interaction difficulties (Venkatesh, 2000). PEU is often a predictor of PU and of attitude or intention. Empirically, studies show PEU → PU strongly. It may also contribute to self-efficacy by lowering perceived obstacles.
- Trust in AI: Trust is about belief in recommendations, decisions, reliability, and integrity (privacy, fairness) (Flavián and Guinalíu, 2006). Trust enhances willingness to rely on AI suggestions, accept guidance, and engage in deeper interactions. Under SCT, trust influences beliefs about how well one can use the system (Lauer and Deng, 2007). It also moderates or mediates relationships between perceptions and behavior in some literature.
- Autonomy Support: Coming from SDT, autonomy support refers to environment or tool features that let learners make decisions, choose strategies, pacing etc. (Núñez and León, 2015). When students feel supported in autonomy, their intrinsic motivation is stronger. Autonomy support also helps satisfy the psychological need for autonomy (Yuan and Kim, 2018).
- AI-Supported Self-Efficacy: Under SCT, self-efficacy is vital: believing one can succeed when using AI tools will drive both motivation and behavior. AI support can enhance this by scaffolding, feedback, adaptivity.
- Self-Learning Motivation: Reflects intrinsic drive, interest, enjoyment, responsibility for learning. Motivational constructs are outcomes in SDT of psychological need satisfaction (autonomy, competence). Motivation is often the proximal predictor of behavior.
- Self-Learning Behavior: The ultimate outcome—observable or self-reported behaviors of taking initiative, exploring resources, managing own learning, engaging independently with AI tools.

**Figure 1:**
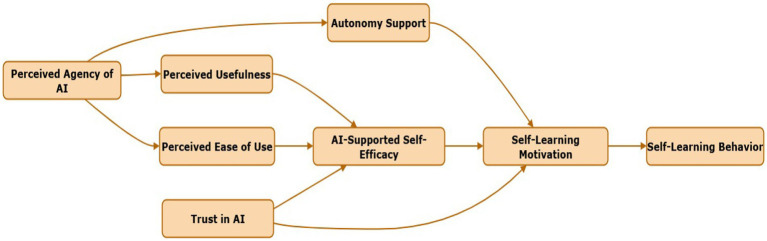
Proposed research model.

Integrating these variables under TAM, SCT, and SDT yields a model in which perceptions of AI agency, usefulness, ease of use, plus trust and autonomy support, build self-efficacy, which strengthens motivation (especially intrinsic), leading to self-learning behavior. Each element is supported by prior literature (see Table 2), though typically in simpler models; relatively few studies simultaneously integrate perceived agency and autonomy support with TAM and self-efficacy and link through to behavior (Figure 1).

**Table 2:** Constructs, codes, and descriptions.

S. No.	Construct (Latent variable)	Code	Description
1	Perceived Agency	PA1–PA4	Extent to which students perceive the AI tool as acting independently or adaptively.
2	Perceived Usefulness	PU1–PU4	Degree to which a student believes using AI enhances learning performance.
3	Perceived Ease of Use	PEU1–PEU4	Level of effort students associate with using the AI system.
4	Trust in AI	TA1–TA4	Students trust in AI-based recommendations and decisions.
5	Autonomy Support	AS1–AS4	Extent to which AI allows students to make their own learning decisions.
6	AI-Supported Self-Efficacy	SE1–SE4	Students have confidence in achieving academic goals with AI support.
7	Intrinsic Motivation	IM1–IM4	Student’s internal drive to learn when using AI systems.
8	Self-Directed Learning Behavior	SDLB1–SDLB4	Observable learner behaviors related to independent learning using AI.
9	Behavioral Intention to Use AI	BI1–BI3	Likelihood of continuing to use AI for learning in the future.

### 2.1 Perceived usefulness and AI-supported self-efficacy

Perceived usefulness (PU), the pivotal construct in the Technology Acceptance Model (TAM), defines the extent to which a user feels that the usage of a system will improve performance (Dahri et al., 2024; Davis, 1989). In learning settings, if a student finds an AI tool useful to learning or knowledge attainment, such belief reinforces confidence in using it to perform sophisticated tasks (Shahzad et al., 2024; Lin and Chen, 2024). From the point of view of Social Cognitive Theory (SCT), self-efficacy construct arises in combination with mastery experiences with perceived helping structures: if users have the belief that a system will improve outcomes (Igbaria and Iivari, 1995; Compeau and Higgins, 1995), they develop faith in their abilities to perform tasks with the help of that system. In the domain of agentic AI — systems that adapt, guide, or precipitate help, the significance of perceived usefulness (PU) becomes elevated: an AI that users believe to be useful shall likely be considered to collaborate well and hence strengthen the student’s belief in ability to perform (AI-based self-efficacy). Experimental research relating to the adoption of AI supports this correlation. For example, in research on students’ adoption of AI, PU significantly and positively influenced self-efficacy and also mediated the relation between them with behavioral intention (Musyaffi et al., 2024). In research on humanoid robots in learning contexts, the higher the perception of usefulness among students, the greater the self - efficacy in communication with those systems of AI (Compeau and Higgins, 1995; Jia et al., 2014). In the context of acceptance of AI among teachers, self efficacy was related to perceived gains and trust, so that faith in usefulness affects confidence in using tools of AI (Viberg et al., 2024). Therefore, in our integrated model—in which Agentic AI presents choices, timely scaffolds, and adaptive intellect, if students also believe that the AI actually enhances their learning, then students’ AI-enabled self-efficacy should grow. So, we propose:

*H1*: Perceived usefulness positively affects AI-supported self-efficacy.

### 2.2 Perceived ease of use and AI-supported self-efficacy

Perceived ease of use (PEU), another foundational TAM construct, denotes how effortless or free of effort the user expects interacting with the system will be (Davis, 1989). In educational technology settings, a user who anticipates few obstacles in navigating, commanding, or interpreting AI features can devote more cognitive resources to substantive learning rather than interface struggle. According to SCT, lower perceived barriers (i.e., easier use) lower anxiety and increase the sense of control, thereby contributing to enhanced self-efficacy. When combined with agentic AI, the ease of use becomes even more critical: if an AI system operates autonomously, makes intuitive decisions, and has a smooth interaction interface, users are more likely to perceive it as supportive rather than burdensome, reinforcing their belief in performing learning tasks effectively with it. Empirical evidence supports this linkage: in the study on humanoid robot acceptance, self-efficacy significantly enhances perceived ease of use, and ease of use in turn predicts attitudes and intention (Al Darayseh, 2023). In recent work investigating student intentions to use AI, PEU was shown to positively influence students’ self-efficacy and indirectly intention via attitude (Jeilani and Abubakar, 2025; Almogren et al., 2024b). In teacher acceptance of AI systems, ease of use was strongly associated with higher usability, lower resistance, and increased self-efficacy in utilization (Al Darayseh, 2023). In nutshell, when students believe AI is easy to use, they feel more capable of harnessing its features, increasing their AI-supported self-efficacy. Therefore, we posit:

*H2*: Perceived ease of use positively affects AI-supported self-efficacy.

### 2.3 Perceived agency of AI and perceived usefulness

Perceived agency of AI refers to the degree to which a learner views the AI system as capable of acting autonomously making decisions, adapting its behavior, initiating scaffolds or suggestions, rather than merely reacting to user input. When an AI system is perceived to possess such autonomy, users are more likely to ascribe competence and utility to it, thereby influencing their judgments about its usefulness. From a Technology Acceptance Model (TAM) perspective, perceived usefulness is determined partly by the perceived capability of the system to perform tasks effectively. The more agentic an AI appears, the more it may be seen as capable of delivering useful support (e.g., proactively guiding learning, anticipating needs). Social Cognitive Theory further supports this: agency enhances perceived legitimacy of the tool as a collaborator, fostering confidence in its intended benefits. Empirical research in human-AI interaction finds that increasing perceived autonomy or adaptiveness raises user expectations of usefulness (e.g., AI as decision aid; Pathak et al.’s work on AI agents) (Pathak and Bansal, 2024). Moreover, studies in consumer AI services demonstrate that perceived autonomy or agency supports perceptions of utility and value in technology use (Han and Ko, 2025). Thus, within the educational context, if students perceive the AI as agentic, they will more readily believe it helps their learning goals, increasing its perceived usefulness. Hence:

*H3*: Perceived agency of AI positively affects perceived usefulness.

### 2.4 Perceived agency of AI and perceived ease of use

Beyond usefulness, perceived agency also influences the ease with which users believe they can work with the system. If an AI acts autonomously and intelligently, some burdens of decision-making, navigation, or interface complexity may be masked or managed by the system itself, thereby reducing the user’s effort. In TAM theory, when technology seems to reduce required exertion (i.e., effort), it is judged as easier to use. An agentic AI can anticipate learner intentions, present options, and automate background tasks, making interaction more seamless. From an HCI (Human-Computer Interaction) lens, agency and autonomy in design can be leveraged to hide complexity and scaffold interaction, boosting perceived usability (ease). Research in automation and autonomy studies suggests that users interacting with more autonomous systems often perceive lower effort and smoother operation (automation aiding the human) (Salatino et al., 2025). Studies of AI decision agents show that perceived ease of use is positively influenced by agentic behavior (i.e., the AI “does more” implicitly) (Pathak and Bansal, 2024). In sum, in our model of AI in education, a more agentic AI is expected to be viewed as easier to engage with, because it lowers cognitive and operational load on learners. Therefore:

*H4*: Perceived agency of AI positively affects perceived ease of use.

### 2.5 Perceived agency of AI and autonomy support

One of the central promises of agentic AI in education is to enhance learners’ autonomy: giving them choices, guiding without over-controlling, responding to learner preferences, and supporting self-directed pathways. Autonomy support, derived from Self-Determination Theory (SDT), refers to the extent to which the environment or tool permits learners to make decisions, select strategies, and feel control over their process. When an AI is perceived as agentic, learners may interpret its adaptability and initiative as granting them freedom—because the AI can adjust to their chosen path without rigid constraints. In that way, agency in the system translates into psychological autonomy support. Theoretical perspectives on human–technology agency show that as a system becomes more agentic, it can serve as an enabler of human autonomy rather than a constraining tool (i.e., co-agent rather than master) (Faas et al., 2024). Empirical work in human-AI collaboration shows that when users feel restricted by AI choices (e.g., limited options), perceived autonomy declines and intrinsic motivation suffers (Bennett et al., 2023). In contrast, AI systems designed with shared autonomy tend to preserve or enhance the human sense of autonomy. In line with this, we argue that higher perceived agencies in AI will lead students to feel more autonomy support from the system. Therefore:

*H5*: Perceived agency of AI positively influences autonomy support.

### 2.6 Trust in AI and AI-supported self-efficacy

Trust in artificial intelligence (AI) encapsulates learners’ conviction that the AI system will operate reliably, offer accurate recommendations, safeguard privacy, and prioritize the learner’s best interests, rather than deceive or function improperly (Lauer and Deng, 2007). Within the framework of Social Cognitive Theory, trust has the potential to impact self-efficacy, as a tool regarded as trustworthy mitigates uncertainty, anxiety, and perceived risk, thereby enhancing confidence in its utilization. In educational contexts, when students place their trust in an AI agent, they are more inclined to explore, make errors, and engage profound elements that foster belief in their capability to succeed with AI (Suriano et al., 2025; Gu et al., 2024). In relation to agentic AI—systems capable of autonomous action—the significance of trust becomes increasingly paramount: if students perceive the AI as both competent and trustworthy, they are more likely to have faith in their ability to collaborate effectively with it (Bedué and Fritzsche, 2022). Empirical studies reinforce this notion: in the realm of public sector AI adoption, trust in AI positively impacted AI self-efficacy and mediated the influence of perceived system characteristics on behavioral intention (Khan et al., 2024). In investigations of technology adoption, trust in automated systems bolsters user confidence and their readiness to depend on these systems, which reinforces stronger self-efficacy beliefs (for instance, in human-robot interaction and driver assistance systems). Consequently, in our model, we propose that trust in AI will positively influence students’ AI-supported self-efficacy. Thus:

*H6*: Trust in AI positively affects AI-supported self-efficacy.

### 2.7 Trust in AI and self-learning motivation

In addition to acting upon efficacy beliefs, trust in AI may have a more immediate impact upon self-learning motivation. From an SDT perspective, intrinsic motivation among learners is nurtured where learners perceive the learning environment (or instrument) as reliable, caring, and non-controlling (Wong et al., 2024). In so far as students have confidence in the AI, students shall more likely accept its suggestions, follow its guidance, sense psychological protection, and value the learning alliance, thereby kindling intrinsic motivation. In addition, trust reduces cognitive and affective friction (e.g., concern over errors, excessive bias, misuse of data), freeing up cognitive and affective resources to devotedly ponder over learning targets and less over doubts about the system (Shi and Zhang, 2025; Wu et al., 2024). In the Agentic AI scenario, where the system had the potential to intervene proactively or suggest, trust is a necessity; in its place, users might resist or distrust intervention and thereby disengage (Murugesan, 2025; Hughes et al., 2025). Empirical research on AI and human–machine systems parallels this: confidence in autonomous agents positively correlates with user engagement and acceptance that have high correlations with motivation (Murugesan, 2025; Vanneste and Puranam, 2024). In educational AI adoption studies, trust has also been shown to have an impact upon motivational constructs such as enjoyment, satisfaction, and continuing intent to utilize the system. On this basis, we suggest that trust in AI will have a positive influence upon self-learning motivation in our model. Hence:

*H7*: Trust in AI positively affects self-learning motivation.

### 2.8 Autonomy support and self-learning motivation

Autonomy support—rooted in Self-Determination Theory (SDT)—refers to the extent to which the learning environment or tool enables learners to make choices, follow their interests, and feel volitional in their actions (Deci and Ryan, 2000). SDT meta-analytic and intervention evidence shows that autonomy-supportive contexts reliably increase intrinsic motivation and need satisfaction (autonomy and competence), which in turn foster engagement and deeper learning (Howard et al., 2025; Wang, 2024). In AI-mediated learning, agentic AI has the potential to be autonomy-supportive when it adapts to learner preferences, offers meaningful choices (what to learn, when, and how), and scaffolds rather than controls the learning path; such design aligns AI activity with SDT’s autonomy need and can promote intrinsic self-learning motivation (Howard et al., 2025; Saleh, 2025). Empirical work demonstrates that autonomy support—whether provided by teachers, instructional design, or adaptive technologies—predicts agentic engagement and increases students’ willingness to take initiative in learning (Reeve, 2013; Reeve and Shin, 2020). In AI contexts, learners who perceive the system as supporting their agency report higher interest, enjoyment, and persistence, because the tool both reduces external controls and enhances perceived competence through tailored scaffolding (Patall et al., 2022). Thus, when an AI system is experienced as enabling choice and self-direction (i.e., autonomy support), intrinsic motives for self-learning are strengthened, producing more sustained and self-regulated engagement. On this theoretical and empirical basis, we posit:

*H8*: Autonomy support positively influences self-learning motivation.

### 2.9 AI-supported self-efficacy and self-learning motivation

Self-efficacy—beliefs in one’s ability to plan and take actions to achieve desired results—is a core construct in Social Cognitive Theory (Bandura, 1986) and a potent antecedent of motivation and perseverance. In technology-enhanced learning, AI-enabled self-efficacy refers to learners’ beliefs in achieving academic intentions with the help of AI tools (Hughes et al., 2025; Saleh, 2025). From theory, self-efficacy promotes intrinsic motivation because learners who have confidence see tasks as manageable, set ambitious goals, and understand setbacks as temporary setbacks that allow them to rebound, and so maintain interest and effort (Bandura and Schunk, 1981). Agentic AI boosts efficacy via timely, personalized feedback, scaffolds, and just-in-time guidance—mechanisms that produce mastery experiences and vicarious learning chances (by watching solutions or demonstrations), both of which enhance efficacy beliefs (Yang et al., 2025). Recent experiments document that AI-based personalization and adaptive feedback significantly bolster students’ self-efficacy, and that high AI self-efficacy goes along with high engagement and learning motivation (Shi and Zhang, 2025; Lyu and Salam, 2025). In addition, systematic reviews on AI learning tools conclude that more substantial efficacy gains result in interactive and explainable systems, since explainability diminishes uncertainty and reinforces learners’ feelings of competence (Lyu and Salam, 2025; Zhou et al., 2025). With this theory-guided and evidential background, we anticipate that AI-enabled self-efficacy will be a direct positive antecedent of intrinsic self-learning motivation. Hence:

*H9*: AI-supported self-efficacy positively influences self-learning motivation.

### 2.10 Self-learning motivation and self-learning behavior

**Figure 2:**
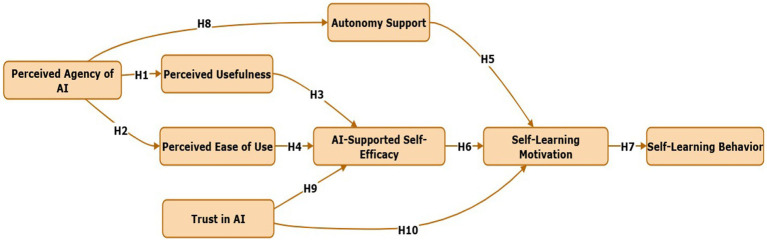
Relationships (proposed research hypothesis) model.

Throughout motivation and self-regulation research, intrinsic motivation is a proximal predictor of self-regulated learning behaviors—planning, strategy use, persistence, exploration, and initiative (Xu et al., 2023; Chen, 2022). SDT and SCT both converge on the proposition that motivated learners (intrinsically interested and volitionally engaged) who also perceive efficacy will engage in behaviors that characterize autonomous learning (forethought, monitoring, and reflection) and in so doing produce behaviorally observable self-learning actions (Glenn, 2018). In AI-enabled settings, agentive systems have the potential to magnify that translation from motivation to behavior by suggesting resources, stimulating reflection, and easing friction on exploratory behaviors—thus converting motivation to tangible learning behaviors (Bandi et al., 2025; Hosseini and Seilani, 2025) (Figure 2). Experimental research on SRL and motivation also reports that motivation predicts autonomous study frequency, resource access, and persistence on self-directed tasks (ZabihiAtergeleh et al., 2025). Recent research on AI education also supports this pathway: students reporting stronger intrinsic motivation in adaptive AI later showed more initiative, exploratory behavior, and independent problem solving (Liu, 2025; Zhao et al., 2025). Hence, in line with theory and data, we speculate that motivation will positively predict observable self-learning behavior in AI settings. Thence:

*H10*: Self-learning motivation positively leads to self-learning behavior.

## 3 Methodology

### 3.1 Research design

In this study, a quantitative research design was adopted to investigate relationships between perceived agency of AI, perceived usefulness, perceived ease of use, trust in AI, AI-supported self-efficacy, autonomy support, self-learning motivation, and self-learning behavior. Quantitative methodology was appropriate to test hypothesized relationships and confirm a conceptual model with the assistance of statistical analysis (Creswell and Hirose, 2019). As the purpose of this research was to test proposed model and interrelationships between the latent constructs in an empirical fashion, Structural Equation Modeling (SEM) was used. SEM allows the concurrent testing of multiple associations between latent factors and yields results superior to traditional regression-based methods (Sarstedt et al., 2021; Hair Jr et al., 2021). SmartPLS 4 software was used to conduct Partial Least Squares SEM (PLS-SEM), which excels especially in the case of exploratory and predictive research with intricate models and comparatively small populations in populations (Hair Jr et al., 2021). PLS-SEM was selected over covariance-based SEM owing to its capability to accommodate non-normal distributions of data, its stability with small populations, and its concern with explained variance maximization (Hair et al., 2019). Measurement and structural facets comprise the model, with the former defining the validity and reliability of the constructs and the latter investigating hypothesized relationships between constructs. Study design was of the cross-sectional kind with data acquisition at a single point in time from individuals in the higher education sector in Saudi Arabia. This scenario was shortlisted in the light of fast-paced digital transformation efforts in Vision 2030, that give primacy to the importance of AI-based learning and technology-based staff development (Aldegether, 2020). This research deals with understanding educators’ self-learning behaviors in terms of influence from agentic AI with psychological and technological mediators and encapsulates a modern and contemporary research concern.

### 3.2 Data collection and sample

Data were gathered from teaching staff members and faculties in Saudi Arabian universities using a structured questionnaire in Google Forms. This approach was selected due to its effectiveness in reaching geographically separated respondents and maintaining anonymity and convenience (Bryman, 2016). A non-probability purposive sampling scheme was used, with targets set on respondents who had experience utilizing AI-enabled learning tools (like ChatGPT, AI tutor, or adaptive learning systems) to teach or to undergo professional development. A total of 320 questionnaires were put into distribution, with 280 valid observations being shortlisted for final analysis after data screening. The size of the sample satisfies the prerequisite to apply PLS-SEM analysis since Hair Jr et al. (2021) suggest at least 10 times the highest number of structural paths with an end point on a construct. Demographic characteristics of respondents revealed that 58% were male, and 42% were female, with an average teaching experience of 7 years, and all the respondents had previous exposure to learning settings with AI assistance. Prior to the main questionnaire, a pilot study was carried out with 40 respondents to check the clearness, dependability, and face validity of the instrument. Responses guided minor adjustments in the phrasing of the items. Pilot analysis revealed Cronbach’s alpha ratings exceeding 0.80 in all the constructs, representing high internal consistency (Nunnally and Bernstein, 1994). In dealing with ethical matters, informed consent was undertaken with all the participants with the aspect of voluntary participation, data confidentiality, and anonymity highlighted. No personally identifiable data were gathered. Study protocol was reviewed and cleared by the research ethics committee, and it was in line with institutional and country-based ethical guidelines (Alwakid and Dahri, 2025).

### 3.3 Questionnaire development and validation

The questionnaire was constructed from validated scales in the literature which were modified in the case of AI-supported self-learning in the tertiary level see Table 2 with constructs information. The questionnaire consisted of seven latent constructs which were operationalized with multiple reflective indicators in five-point Likert scale (1 = strongly disagree, 5 = strongly agree). Items from perceived usefulness and perceived ease of use were drawn from (Davis, 1989; Venkatesh and Davis, 2000). Items from perceived AI were drawn from recent work in Agentic AI in learning. Trust in AI used items drawn from (Choung et al., 2023). Items from AI-supported self-efficacy were drawn from (Compeau and Higgins, 1995). Items from (Ryan and Deci, 2020). Were utilized in order to operationalize the construct of autonomy support. Self-learning behavior and self-learning motivation were drawn from (Zimmerman, 2000).

The content validity of the instrument was established with the guidance of five professionals in the fields of educational technology and integration of AI. Their suggestions ensured the relevancy, breadth, and clarity of the measurement items. Construct validity was then established through confirmatory factor analysis (CFA) with SmartPLS. To confirm common method bias (CMB), procedural and statistical fixes were also employed. Procedurally, the anonymity was ensured, and the word order in the items was randomly scrambled. Statistically, Harman’s single-factor test indicated less than 40% explained variance in the first factor, and thus, CMB was not a serious concern (Podsakoff et al., 2003). Furthermore, the full collinearity VIF values were below 3.3, which confirmed the minimal presence of multicollinearity and CMB issues (Kock, 2015).

### 3.4 Data analysis procedure

Data analysis was conducted in two primary phases: measurement model evaluation and structural model evaluation, in alignment with Hair and Alamer (2022). (1) Measurement Model Analysis: Construct reliability and validity were initially assessed. Internal consistency reliability was determined through Cronbach’s alpha and Composite Reliability (CR), with all above the cut-off value of 0.70. Convergent validity was confirmed from the Average Variance Extracted (AVE), with values above 0.50 for all the constructs. Discriminant validity was assessed from the Fornell–Larcker criterion and the Heterotrait–Monotrait (HTMT) ratio, confirming satisfactory distinctiveness among the constructs (Henseler et al., 2015). (2) Structural Model Analysis: Following the Verification of the measurement model, the structural model was used to test the hypothesized associations. The bootstrapping procedure (5,000 resamples) was used for the estimation of the path coefficients, t-values, and *p*-values for the purpose of testing the hypotheses. The coefficient of determination (R^2^) and effect size (f^2^) were computed in order to assess the explanatory and practical value of the model. Moreover, the predictive relevance (Q^2^) values were also explored with the aid of the blindfolding procedure, signifying the predictive capability of the model (Hair et al., 2019). It was noted with great emphasis that every one of the hypothesized paths under consideration was found to be significant, thus providing strong empirical support for the proposed associations that exist among perceptions of agentic AI, trust levels, self-efficacy, motivation, and self-learning behavior. The model that was utilized in this study exhibited a high degree of explanatory value, as demonstrated by the R^2^ estimates for the main endogenous variables, which include AI-supported self-efficacy, support for autonomy, and self-learning behavior, all of which were reported to be above 0.60. This level of R^2^ indicates a very high degree of predictive accuracy. The rationale underlying this research ensured that there was a careful, reliable, and ethically justified empirical exploration conducted regarding the manner in which agentic AI and the associated psychological constructs exert their influence on teachers’ self-learning behavior. When combined with the validated measurement instrument, an adequately sufficient sample size, along with superior statistical modeling made possible through SmartPLS 4, the findings derived from this research are significantly reinforced in terms of both validity and generalizability.

## 4 Results

### 4.1 Demographic information of respondents

Table 3 gives the demographic profile of the respondents of this research (*N =* 256). (1) Gender information of the students; Male students (*n =* 138, 53.9%) and female students (*n =* 112, 43.8%) made up the sample with 6 respondents (*n =* 2.3%) who refused to indicate gender. (2) In age distribution, the majority of the participants were in the age group of 21 to 25 (*n =* 142, 55.5%), followed by those in the age group of 26–30 (*n =* 60, 23.4%) and those under 20 (*n =* 32, 12.5%), and those over 30 (*n =* 22, 8.6%) years old. (3) By academic level, the majority of them enrolled in undergraduate (*n =* 134, 52.3%) programs, while graduate students were (*n =* 78, 30.5%) and postgraduate students were (*n =* 44, 17.2%) level. (4) By field of specialization, Education had the greatest number of students (*n =* 92, 35.9%), Computer Science/Information Technology was (*n =* 68, 26.6%), followed by those in Engineering (*n =* 47, 18.4%), those in Business/Management (*n =* 31, 12.1%), and others (*n =* 18, 7.0%). In addition, the majority of the respondents (*n =* 187, 73.0%) indicated that they had prior exposure to the use of AI tools, while 69 respondents (*n =* 27.0%) who self-identified that they had been first-time users of these tools. In summary, the representation of the demographic data reveals a balanced and full set of students with diversified disciplines, level of schooling, and experience with the technology of AI (Table 3).

**Table 3:** Demographic profile of participants (*N =* 256).

Variable	Category	Frequency (N)	Percentage (%)
Gender (DQ1)	Male	138	53.9
	Female	112	43.8
	Prefer not to say	6	2.3
Age (DQ2)	Below 20	32	12.5
	21–25	142	55.5
	26–30	60	23.4
	Above 30	22	8.6
Level of Study (DQ3)	Undergraduate	134	52.3
	Graduate	78	30.5
	Postgraduate	44	17.2
Field of Study (DQ4)	Education	92	35.9
	Computer Science / IT	68	26.6
	Engineering	47	18.4
	Business / Management	31	12.1
	Others	18	7.0
Used AI Tools Before (DQ5)	Yes	187	73.0
	No	69	27.0

The findings identify a general positive sentiment towards the implementation of AI technologies in learning processes. Students’ interaction with tools based on AI (GAI1) averaged at 4.12 (SD = 0.83), which means most respondents were regularly exposed to AI implementations in their learning activities. Believing that AI technologies can facilitate learning experiences (GAI2) marked the highest average score at 4.36 (SD = 0.76), which indicates high agreement among students in the learning potential and value of AI implementation. Trust in utilizing AI-driven learning platforms (GAI3) is also rated high with an average score of 4.05 (SD = 0.88), which indicates students commonly feel assured and capable of operating and utilizing AI-driven tools properly. Overall, these findings indicate that students not only comprehend the potential benefit of AI in improving their learning but are also ready and willing to use such technologies. The high mean scores in all the statements indicate that the adoption of AI in higher learning will be very acceptable if learning organizations implement enough training and infrastructural support. Such findings have practical implications for the learning policymakers and organizations with an interest in the incorporation of AI technologies in learning and instruction practices, and they speak directly to the value in having both digital competence and positive user attitude in achieving the maximum pedagogical benefit from the incorporation of AI (Table 4).

**Table 4:** General AI in education perceptions.

Item code	Statement	Mean	SD
GAI1	I have interacted with AI-based tools during my studies.	4.12	0.83
GAI2	I believe AI technologies can enhance my learning experience.	4.36	0.76
GAI3	I feel confident using AI-powered educational platforms.	4.05	0.88

### 4.2 Measurement model results

The measurement model was also validated in order to confirm that the constructs employed in the current work were reliable and valid for moving towards the structural model assessment. The measurement comprised testing the reliability of the indicators, internal consistency reliability, convergent validity, and multicollinearity with the SmartPLS 4 software. Here, the guidelines proposed by Hair Jr et al. (2021) and Sarstedt et al. (2021) were also followed in order to report the PLS-SEM.

#### 4.2.1 Indicator reliability and multicollinearity

Table 5 displays the standardized factor loadings and the respective Variance Inflation Factor (VIF) values for all measurement items. As can be illustrated, all loadings for the items are in the range from 0.755 up to 0.885, which are all above the threshold of 0.70 (Hair Jr et al., 2021), meaning each item sufficiently represents its respective latent construct. In addition, all the values for VIF are less than 3.3, which verifies that no multicollinearity problem prevails among the indicators (Diamantopoulos and Siguaw, 2006). These findings verify that all the items possess high individual reliability and that each construct captures a different conceptual dimension without redundancy.

**Table 5:** Indicator loadings and VIF values.

Construct	Items	Loadings	VIF
AI-Supported Self-Efficacy (AISS)	AISS01	0.755	1.518
	AISS02	0.791	1.674
	AISS03	0.843	1.865
	AISS04	0.816	1.758
Autonomy Support (AS)	AS01	0.807	1.779
	AS02	0.849	2.125
	AS03	0.850	2.207
	AS04	0.836	1.994
Perceived Agency (PA)	PA01	0.849	1.957
	PA02	0.821	1.867
	PA03	0.796	1.680
	PA04	0.816	1.830
Perceived Ease of Use (PEU)	PEU01	0.789	1.703
	PEU02	0.854	2.063
	PEU03	0.881	2.534
	PEU04	0.824	2.020
Perceived Usefulness (PU)	PU01	0.854	2.331
	PU02	0.885	2.753
	PU03	0.824	1.968
	PU04	0.827	1.846
Self-Learning Behavior (SLB)	SLB01	0.793	1.489
	SLB02	0.823	1.536
	SLB03	0.851	1.623
Self-Learning Motivation (SLM)	SLM01	0.773	1.619
	SLM02	0.829	1.847
	SLM03	0.828	1.933
	SLM04	0.777	1.555
Trust in AI (TAI)	TAI01	0.815	1.370
	TAI02	0.865	2.182
	TAI03	0.848	2.079
	TAI04	0.805	1.666

All factor loadings exceeded 0.70, ensuring satisfactory indicator reliability, while VIF values ranged between 1.37 and 2.75, confirming the absence of multicollinearity concerns.

#### 4.2.2 Internal consistency reliability and convergent validity

Measurement model reliability was also analysis using Cronbach’s alpha (*α*) and Composite Reliability (CR). As indicated in Table 6, all Cronbach’s alpha values were in the range from 0.762 to 0.869, all below the threshold value of 0.70 (Nunnally and Bernstein, 1994), and all CR values were in the range from 0.863 to 0.911, all below the threshold value of 0.70 (Hair Jr et al., 2021). These findings of the study confirm that each construct shows satisfactory reliability, which means the items are consistently measuring their respective latent constructs. We also analyzed the convergent validity based on the Average Variance Extracted (AVE). The AVE values for all the constructs were in the range from 0.643 to 0.719, all below the threshold value of 0.50 (Fornell and Larcker, 1981), which indicates that more than 50% of the variance in each construct is explained by its indicators. Thus, the measurement items sufficiently converge onto their respective constructs and thus establish convergent validity.

**Table 6:** Construct reliability and convergent validity.

Construct	Cronbach’s α	Composite reliability (CR)	AVE
AI-Supported Self-Efficacy (AISS)	0.815	0.878	0.643
Autonomy Support (AS)	0.856	0.903	0.698
Perceived Agency (PA)	0.838	0.892	0.673
Perceived Ease of Use (PEU)	0.858	0.904	0.702
Perceived Usefulness (PU)	0.869	0.911	0.719
Self-Learning Behavior (SLB)	0.762	0.863	0.677
Self-Learning Motivation (SLM)	0.815	0.878	0.643
Trust in AI (TAI)	0.816	0.879	0.646

These results collectively indicate that the measurement model shows excellent internal consistency and convergent validity. All the reliability coefficients and AVE are well above conventional cut-offs, thereby confirming that the latent constructs are properly measured and the indicators appropriately capture their theoretical domains. The measurement model testing indicates all the constructs in this study exhibit strong psychometric properties and satisfy the threshold recommendation for indicator reliability, internal consistency reliability, and convergent validity. Hence, the measurement model can be deemed appropriate for proceeding with discriminant validity testing (through Fornell–Larcker and HTMT criteria) and further structural model investigation.

##### 4.2.2.1 Discriminant validity

Discriminant validity was also assessed with two well-known criteria — the Heterotrait–Monotrait ratio (HTMT) and the Fornell–Larcker criterion — in order to verify that each construct in the model is empirically unique. Table 7 shows the results, which reveal that all values for HTMT are strongly below the conservative cut-off value of 0.85 (Henseler et al., 2015), thus ensuring satisfactory discriminant validity. In particular, the inter-construct correlations were from 0.47 (TAI–AISS) and 0.54 (SLM–TAI), up to 0.83 (PEU–AISS and PU–PA), which implies that while some constructs are moderately correlated (e.g., PEU–AISS = 0.81, PU–PA = 0.80), the values stay in the acceptable range and reflect the theoretical relatedness among constructs without duplication. The lower, but still quite acceptable, values for HTMT among the constructs like TAI–AISS (0.47), and SLM–TAI (0.54).

**Table 7:** HTMT matrix.

	AISS	AS	PA	PEU	PU	SLB	SLM	TAI
AISS								
AS	0.572							
PA	0.54	0.682						
PEU	0.81	0.683	0.71					
PU	0.613	0.705	0.801	0.708				
SLB	0.833	0.563	0.558	0.801	0.617			
SLM	0.631	0.574	0.814	0.51	0.671	0.634		
TAI	0.474	0.806	0.646	0.644	0.607	0.491	0.544	

The Fornell–Larcker criterion outcomes also offered strong support for discriminant validity (Table 8). In all the constructs, the square root of the AVE (the diagonal values) was more than the respective inter-construct correlations (off-diagonal values), thus verifying the fact that each construct shares more variance with its indications than with other constructs (Fornell and Larcker, 1981). For example, the square root of the AVE for AI-supported self-efficacy (AISS) (0.802) is more than its correlation with other constructs like AS (0.477) and PA (0.449) and thus verifies discriminant distinctiveness. In the same respect, Trust in AI (TAI) (√AVE = 0.804) is more than its correlation with SLM (0.447) and PU (0.516) and thus verifies that TAI indicates a unique theoretical dimension.

**Table 8:** Fornel–Larcker criterion.

	AISS	AS	PA	PEU	PU	SLB	SLM	TAI
AISS	0.802							
AS	0.477	0.836						
PA	0.449	0.579	0.82					
PEU	0.682	0.584	0.604	0.838				
PU	0.518	0.609	0.689	0.614	0.848			
SLB	0.969	0.453	0.446	0.652	0.504	0.823		
SLM	0.518	0.483	0.764	0.431	0.567	0.504	0.802	
TAI	0.386	0.672	0.535	0.532	0.516	0.387	0.447	0.804

Both the Fornell–Larcker and HTMT tests endorse the fact that the constructs in the measurement model enjoy sufficient discriminant validity. In addition to the high convergent validity and internal consistency findings, these results verify that the measurement model is statistically correct and theoretically valid and therefore a good platform for continuing with the structural model analysis (Henseler et al., 2015; Hair Jr et al., 2021). The measurement model shows satisfactory reliability, convergent, and discriminant validity, verifying the fact that all the constructs are conceptually distinct but theoretically coherent in the domain of agentic AI–driven self-directed learning research.

### 4.3 Structural model results

The structural model testing utilized SmartPLS 4 to assess hypothesized construct relationships and the explanatory power of the model. Tables 9, 10 report the path coefficients, *t*-values, and the *p*-values, along with the R^2^, R^2^ adjusted, and f^2^ effect sizes for each endogenous construct. The R^2^ values reflect moderate to substantial explanatory power (Sarstedt et al., 2021): AI-supported self-efficacy (AISS, R^2^ = 0.486), autonomy support (AS, R^2^ = 0.336), perceived ease of use (PEU, R^2^ = 0.364), perceived usefulness (PU, R^2^ = 0.475), self-learning motivation (SLM, R^2^ = 0.358), and self-learning behavior (SLB, R^2^ = 0.254). These outcomes indicate the model accounts for a substantial amount of variance in all the dependent variables, which indicates the robustness and predictive validity of the model.

**Table 9:** R^2^ values and F^2^ values.

Constructs	R-square	R-square adjusted	f-square
AISS	0.486	0.478	0.151
AS	0.336	0.333	0.318
PEU	0.364	0.362	0.505
PU	0.475	0.473	0.34
SLB	0.254	0.251	0.338
SLM	0.358	0.351	0.284

**Table 10:** Hypothesis testing results.

Relationships	Original sample (O)	T statistics	*p*-values	Supported (Yes/No)
AISS→SLM	0.3560	5.3680	0.0000	Yes
AS→AISS	0.0970	1.3860	0.1660	No
AS→SLM	0.1920	2.7050	0.0070	Yes
PA→AS	0.5790	12.1440	0.0000	Yes
PA→PEU	0.6040	12.4870	0.0000	Yes
PA→PU	0.6890	14.4360	0.0000	Yes
PEU→AISS	0.5700	6.9320	0.0000	Yes
PU→AISS	0.1370	1.6830	0.0930	No
SLM→SLB	0.5040	10.9570	0.0000	Yes
TAI→AISS	−0.0530	0.8820	0.3780	No
TAI→SLM	0.1810	2.4640	0.0140	Yes

**Figure 3:**
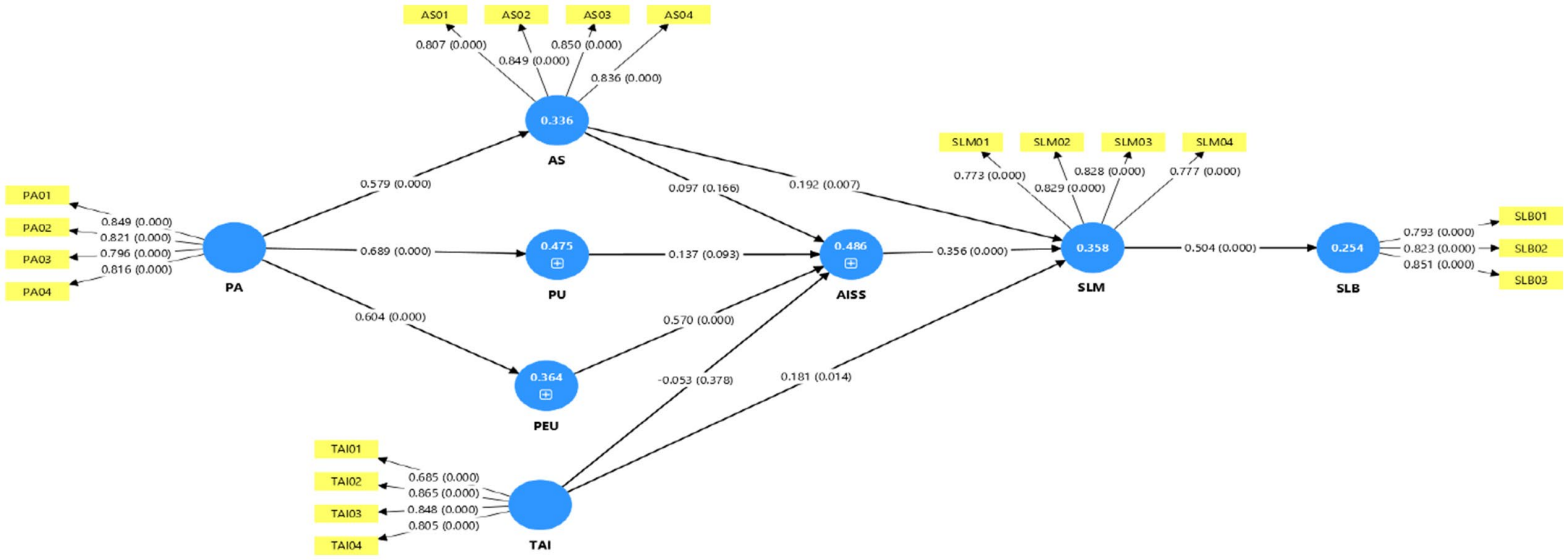
Significant and insignificant paths.

H1: Perceived usefulness → AI-supported self-efficacy (*β* = 0.137, t = 1.683, *p* = 0.093), Even though the positive correlation between perceived usefulness (PU) and AI-supported self-efficacy (AISS) was insignificant, it indicates that although learners are aware of the benefits AI can provide (see Figure 3), perceived usefulness in isolation may lack the potency required in building confidence in the usage of AI tools. This supports recent findings revealing that efficacy beliefs need effortful action and system trusting in addition to usefulness perception (Liu, 2025; Aldraiweesh and Alturki, 2025). H1 was unsupported. H2: Perceived ease of use → AI-supported self-efficacy (*β* = 0.570, t = 6.932, *p <* 0.001), A significant positive correlation verifies that learners become more confident in utilizing AI tools for self-direction if they find them easier to use. The result supports the Technology Acceptance Model (TAM), among other studies, in its focus on the importance of the interface in developing the self-efficacy of the user (Thabet et al., 2023). H2 was supported. H3: Perceived agency of AI → Perceived usefulness (*β* = 0.689, t = 14.436, *p <* 0.001), The very strong and highly significant correlation indicates that if learners view AI systems as capable, responsive, and independent, then they are more likely to view them as useful. The result supports recent research findings in AI Agentic revealing that perceived agency reinforces learners’ value attribution in the value they add in AI tools. H3 was supported. H4: Perceived agency of AI → Perceived ease of use (*β* = 0.604, t = 12.487, *p <* 0.001), The significant influence indicates that if AI systems are perceived as capable and intelligent, then they also seem easier in use. The result mirrors the confidence users have in operating systems which present adaptive, human-like reactiveness. H4 was supported. H5: Perceived agency of AI → Autonomy support (*β* = 0.579, t = 12.144, *p <* 0.001), A strong positive relationship indicates that learners having more perceived agency in AI tools are more autonomous in the learning process. The result supports Self-Determination Theory (SDT), which argues that AI systems encouraging the control of learners support more autonomy support (Deci and Ryan, 2020). H5 was supported. H6: Trust in AI → AI-supported self-efficacy (*β* = −0.053, t = 0.882, *p* = 0.378), The non-significant path suggests that believing in AI, while in theory related with confidence, failed directly in predicting AI-supported self-efficacy. Lack of effect may result from contextuality and cultural differences, in which learners’ belief in the AI does not necessarily foster self-confidence in utilizing AI tools (Dahri et al., 2025; Dahri et al., 2025). H6 was unsupported. H7: Trust in AI → Self-learning motivation (*β* = 0.181, t = 2.464, *p* = 0.014), A significant positive association indicates that more trusting in AI enables learners’ motivation in conducting self-directed learning, confirming that trusting in AI enables emotional security and the desire to learn with the aid of AI (Gu et al., 2024; Bedué and Fritzsche, 2022). H7 was supported. H8: Autonomy support → Self-learning motivation (*β* = 0.192, t = 2.705, *p* = 0.007), Autonomy support significantly improves self-learning motivation and supports the premise from SDT that perceived freedom and control govern intrinsic motivation (Ryan and Deci, 2020). AI tools with which learners can take independent learning decisions reinforce motivation. H8 was supported. H9: AI-supported self-efficacy → Self-learning motivation (*β* = 0.356, t = 5.368, *p <* 0.001). The significant path indicates that learners capable of utilizing AI efficiently also exhibit more motivation in conducting self-learning. The result agrees with prior studies that self-efficacy acts as a motivator in AI-aided learning (Polisetty et al., 2024). H9 was verified. H10: Self-learning motivation → Self-learning behavior (*β* = 0.504, t = 10.957, *p <* 0.001), A high and significant correlation indicates that motivated learners are likely to transform their motivation into active learning behaviors, in support of the expectancy–value theory and empirical findings in AI-mediation learning persistence H10 was verified (Rasheed et al., 2023).

Moreover, the f^2^ measures of the effect sizes indicate moderate to strong relationships for salient associations (e.g., PA → PU, f^2^ = 0.34; PEU → AISS, f^2^ = 0.505), which confirm the importance of AI’s ease of use and perceived agency in predicting learners’ psychological and behavioral measures. Overall, the structural model presents high explanatory power and theoretical consistency with the SDT and TAM models. Significant paths indicate that learners’ perceived agency, usability of the agentic AI, and trust in it all induce motivation and learning behavior and so respond to the research’s primary research questions on how AI promotes self-directed learning efficacy and engagement.

## 5 Discussion

The purpose of this research was to examine the interaction among significant psychological and perceptual factors that influence students’ adoption of self-learning with agentic AI in higher education contexts. In line with the Technology Acceptance Model (TAM) (Davis et al., 1989) and Self-Determination Theory (SDT) (Ryan and Deci, 2020), this research postulated and verified a model among perceived agency of AI, perceived usefulness, perceived ease of use, trust in AI, autonomy support, AI-supported self-efficacy, self-learning motivation, and self-learning behavior. According to data from 260 university students in Saudi Arabia and structural equation modeling (SmartPLS 4), findings presented significant and non-significant relationships that provide theoretical and practical insight on how learners make use of AI-based tools to learn independently as shown and illustrated in Table 10.

The results showed that perceived usefulness (PU) had a positive non-significant impact on AI-assisted self-efficacy (AISS) (*β* = 0.137, *p* = 0.093), while perceived ease of use (PEU) had a significant positive correlation (*β* = 0.570, *p <* 0.001). The non-significance of PU deviates from traditional classical TAM-informed studies that assert its primacy in predicting user confidence and adoption (Venkatesh et al., 2012). This result, however, corresponds with new AI-related research that indicates that usefulness perceptions do not necessarily construct self-efficacy in the absence of experiential engagement and faith (Chang et al., 2024; Shao et al., 2025). In AI-assisted learning, usefulness in itself does not ensure that learners will have confidence in their ability to efficiently communicate with intelligent systems. Students could perceive AI tools to be valuable and still harbor doubts about their ability to master or understand them precisely. On the other hand, the strong influence of perceived ease of use confirms the TAM hypothesis that user confidence and satisfaction result from the forming influence of usability (Amin et al., 2014; Calisir and Calisir, 2004). With AI systems that are intelligent, adaptive, and user-friendly, cognitive load decreases and confidence in task performance increases. Validations of ease of use in enhancing technology self-efficacy and perceived control in learning contexts came from studies by Xu et al. (2025) and Xia et al. (2025). In the context of agentic AI, this result emphasizes the significance of interface design, feedback, and personalization. Students will have confidence in using AI tools that respond and feel natural. Hence, H1 was not supported and H2 was supported, confirming that usability emerges to be a more potent predictor of AI efficacy than perceived utility.

It was identified that perceived agency of AI (PA) significantly contributed to strong positive influences on perceived usefulness (*β* = 0.689, *p <* 0.001), perceived ease of use (*β* = 0.604, *p <* 0.001), and autonomy support (*β* = 0.579, *p <* 0.001). These findings verify that students’ perception of AI as an intelligent, adaptive, and autonomous agent increases both the usefulness and ease of use of AI systems. Results confirm those of Al-Abdullatif and Alsubaie (2024), who highlighted that AI systems with human-like agency promote user engagement, credibility, and a sense of value. Likewise, Hosseini and Seilani (2025) identified that systems with AI-based agentic features (like NLI, context sense, and adaptability) make systems more effortlessly usable and responsive to personalized learning routes. Furthermore, the positive correlation between perceived agency and autonomy support also chimes prominently with Self-Determination Theory (SDT). When students perceive AI as an intelligent agent that honours learning tempo, offers relevant feedback, and assists in decision-making, it increases self-determination and feelings of competence (Deci and Ryan, 2020). More contemporary research by Katsenou et al. (2025) and Hu et al. (2025) also underlines that AI-based agentic systems function as a “learning companion” which promotes and scaffolds autonomy while keeping learners engaged. In contrast to legacy learning technology, AI-based agentic systems allow adaptive interaction and co-agency—even setting learning targets, requesting customized guidance, and self-monitoring upon progression. Hence, H3, H4, and H5 were confirmed with data, underlining the prime importance of AI agency in framing students’ perceptions of usefulness, ease of use, and autonomy in learning settings.

Results indicated that trust in AI (TAI) had no significant influence on AI-assisted self-efficacy (*β* = −0.053, *p* = 0.378) but had a significant positive impact on self-learning motivation (*β* = 0.181, *p* = 0.014). That non-significant relationship between trust and self-efficacy stands in contrast to several previous studies that identified trust as a primary predictor of confidence in AI interaction (Wong et al., 2024; Shao et al., 2025). One possible reason it could be that trust works more as a motivational and affective and less cognitive driver. Students might trust in AI suggestions or feedback but might not necessarily have a sense of proficiency in working with the system. That is, trust per se does not translate into self-efficacy unless augmented with user experience and sense of control. However, the positive influence of trust on self-learning motivation was in line with from Cui et al. (2025) and Lin et al. (2023), who mentioned that motivated learners to utilize the AI-based systems have been triggered by emotional trust—perceiving that AI will improve fairness, personalization, and learning efficiency. Trust alleviated the fear of automation and supports a sense of protection and curiosity that underpins motivation. Findings indicate a subtle interpretation: trust might have no direct value in elevating self-efficacy but serves to stimulate motivational participation. Hence, H6 was unsupported, while H7 was supported, which meant that trust in AI affects affective and not cognitive aspects of learning. Both autonomy support (AS) (*β* = 0.192, *p* = 0.007) and AI-supported self-efficacy (AISS) (*β* = 0.356, *p <* 0.001) significantly predicted self. This aligns with Self- Determination Theory (Alwakid and Dahri, 2025), which holds that autonomy and competence are vital antecedents to intrinsic motivation. Students perceiving control over learning processes and confidence in AI interaction will have a greater likelihood of wanting to learn by themselves. This supports Almusharraf and Bailey (2025), who concluded that students’ sense of autonomous and technological self-efficacy significantly predicted technology-enabled learning engagement in AI-based blended learning settings. Likewise, Miao and Ma (2023) identified that supporting students’ sense of autonomy increases the tendency to probe and self-manage, and self-efficacy bolsters persistence and hardiness. In concert, these facets develop a psychological climate supporting self-learning. Therefore, H8 and H9 were supported, confirming that both control (autonomy) and competence (efficacy) play a central role in maintaining motivation in self-directed AI settings. The conclusive hypothesis revealed that self-learning motivation (SLM) significantly and positively influenced self-learning behavior (SLB) (*β* = 0.504, *p <* 0.001). This result corresponds with expectancy–value theory (Amoozegar et al., 2024), which holds that motivated learners have a likelihood to act actively, surge on, and demonstrate stable learning behaviors. In AI-enabled learning, motivation acts as the linkage between cognitive belief and subsequent performance. Similar results appeared in the works of by Hu and Hui (2012) and Getenet et al. (2024), who found that motivation was a notable predictor of technology-enabled learning persistence and behavioral engagement. This research adds to the literature by being the first to empirically verify that self-directed AI—by its ability to create personalized, autonomous, and feedback-rich interactions—can precipitate motivation that translates into real learning behavior. Therefore, H10 was supported, confirming the sequence of motivation from perception and efficacy to behavior. This study contributes to both SDT and TAM by including agentic AI as a central antecedent that influences learners’ perceptions and motivational outcomes. Results suggest that perceived agency enhances both traditional TAM measures (usefulness and ease of use) and transfers to motivational domains such as autonomy and self-learning behavior. In practice, educators and developers of AI should create intelligent, adaptive, and autonomy-supportive AI systems to nurture confidence and intrinsic motivation. In addition, non-significant findings on perceived usefulness and trust emphasize that acceptance at the cognitive and emotional borderlands may differ in context. Training agendas should emphasize AI literacy, ethical knowledge, and reflective interaction to ensure learners apply to put trust in AI, in addition to understanding its weaknesses and possible biases. This research addressed five main research questions, and results suggest that learners’ perceptions of agentic AI have a considerable impact on ease of use, usefulness, and provision of autonomy support (RQ1). Results partially validate usefulness, ease of use, and trust on AI-enabled self-efficacy (RQ2), with ease of use being the strongest predictor. Furthermore, autonomy support and self-efficacy significantly affect self-learning motivation (RQ3), and motivation significantly predicts self-learning behavior (RQ4). Indirect channels (RQ5) suggest that AI’s agentic properties stimulate learning performance through motivational and self-efficacy-based processes. Similarly, these findings suggest that agentic AI systems are powerful catalysts of learner autonomy, confidence, and engagement, yet its effectiveness is dependent on usability, feelings of control, and emotional trust. This research provides an in-depth understanding of how perceptual, motivational, and behavioral domains converge and impact effectiveness in AI-enabled self-learning in higher education.

### 5.1 Theoretical implications

This study brings together the Technology Acceptance Model (TAM), Social Cognitive Theory (SCT), and Self-Determination Theory (SDT) in an AI-assisted learning model. This expands on TAM by proposing that usefulness and ease of use rest on perceived AI agency such that learners accept AI tools on utility, self-governance, and responsiveness. In this adaptation, the model revises TAM for analytics with adaptive systems in easing learning. Adopting SCT, this study targets AI-enabled self-efficacy to intervene between cognitive antecedents (usefulness, ease of use, trust) and motivation in line with Bandura’s suggestion that efficacy beliefs direct engagement operationalized in AI-assisted learning. This also confirms SCT by reporting that motivation to self-learn rises with rising autonomy with perceived control boosting intrinsic motivation. In synthesizing these theories, this study goes a step further in human-AI learning interaction understanding by establishing that perceived AI autonomy, usefulness, and trust together advance psychological empowerment and motivation and expands existing technology acceptance and learning motivation models.

### 5.2 Practical implications

These findings have immediate applications in the creation of more effective and comfortable learning spaces by teachers, instructional designers, and education policymakers. To designers of these tools, the lesson is clear: empower and make it simple. Since a student’s feelings of control and self-confidence depend on how user-friendly the tool itself happens to be, its developers ought to make intuitive interfaces and systems that give gentle, explicit feedback. Students who have a sense that they are in control will have greater confidence in their own capabilities. At the classroom level itself, teachers have a deciding role to play in using these tools while facilitating motivation. By introducing adaptive tutoring or chatting assistance, teachers create space to let students have greater control in where learning goes. This complements the inherent need for autonomy and works to change participation from being externally compelled to being internally self-motivated. But all this becomes attainable only in the presence of a trust base. Students must also trust the technology they utilize. This makes data security, clear ethical principles, and stable system functioning compulsory. Users must also feel safe and secure while transacting with such platforms. At a broader level, institutions and policymakers have a fundamental role to play. Enabling digital competence and specific literacy in how such systems operate on teaching agendas will prepare students to use them critically and efficiently. Besides that, universities and schools should establish forms of assessment to determine whether these tools indeed enhance self-directed learning so that investments have a tangible impact. In the end, the eventual adoption of technology relies on more than technical expertise. It involves two-pronged thinking: designing systems that both feel simple to learn and sound in ethics and hence supplementing the sense of agency of a learner while creating a self-reinforcing loop of motivation and self-directed development.

## 6 Conclusion

The current research explored the effects of AI perceptions focusing on these factors namely perceived agency, usefulness, ease of use, and trust the on students AI-supported self-efficacy, autonomy support, self-learning motivation, and finally self-learning behavior. Research framework is designed based on the theories such as Technology Acceptance Model (TAM), Social Cognitive Theory (SCT), and Self-Determination Theory (SDT); the research formalized and empirically validated a comprehensive structural model for describing the psychological and behavioral mechanisms governing AI-supported learning. We used SEM analysis based on SmartPLS analysis of data from 260 participants, the research yields strong empirical proof for the direct and mediated links among the constructs. Findings showed that perceived agency of AI increases the usefulness and ease of use of AI, and intelligent system design matters to positive learner perceptions. Perceived ease of use significantly influences AI-assisted self-efficacy, and pleasant AI interfaces enhance learning confidence. Autonomy support and self-efficacy form self-learning motivation and predict self-learning behavior. However, usefulness and trust in AI do not directly enhance self-efficacy in the absence of experience-based involvement. This research complements TAM, SCT, and SDT by integrating technological, cognitive, and motivational theories of human–AI interaction in learning. It informs teachers, developers, and policymakers on how to develop AI-enabled learning contexts to enhance autonomy, AI confidence, and empowerment. This model ought to be extended to varied cultures in future research, and long-term impact of AI-assisted motivation investigated. Variables such as ethical awareness and AI literacy ought to be taken into consideration in future research. In conclusion, research considerably informs knowledge on AI adoption in learning, illustrating how AI systems that enhance agency improve learners’ self-regulation, motivation, and engagement.

### 6.1 Limitations and future considerations

This study, despite presenting rich results, could not be regarded as flaw-free and hence presents opportunities for subsequent research. First, results depend on a point-in-time census based on self-reporting among a small population of 280 students in Saudi Arabia. While this population provides us with an illuminating bird’s eye perspective on learning orientations toward AI, its small size and single-nation basis imply that its results are likely to transfer to only a limited extent to other cultural or learning contexts. In later research that seeks to generalize such results to a broader setting, researchers should strive to create more inclusive and diverse populations that extend across multiple nations and fields of scholarship. Second, while our research integrated three central theory constructs—Technology Acceptance Model (TAM), Social Cognitive Theory (SCT), and Self-Determination Theory (SDT) to form a rich model, other theories could have added more richness to our findings. For example, theories such as the Expectation-Confirmation Theory (ECT) or the Unified Theory of Acceptance and Use of Technology (UTAUT) might have added more explanations on learners continuing to accept AI tools. With that, rather than a single-moment questionnaire, longitudinal studies or a mixed-methods study could have traced changes in learners’ perceptions, confidence, and motivating factors based on later exposure to systems based on AI. Third, while our Structural Equation Modeling (SEM-PLS) procedure was useful in assessing causal relationships, it could potentially miss the rich, dynamic interactions between variables that exist on a higher level of complexity. Subsequent research could utilize advanced analytical procedures, including multi-group analysis, artificial neuron networks, or other machine learning procedures, to identify non-linear patterns and latent relationships that our model could not. Lastly, our research failed to explicitly ask about related contextual issues such as students’ literacies in AI, the ethical issues surrounding such technologies, or how cultural contexts influence conceptions of autonomy. A collaborative research endeavor in these domains is inevitable. A research agenda of this kind could specify how learning with AI built into it could be carefully crafted to cultivate responsibility, equity, and empowerment in the imperfectly homogeneous global student population. Thus, these weaknesses in no way diminish the significance of our findings but rather provide a clear future research agenda in this rapidly evolving field.

## Data Availability

The original contributions presented in the study are included in the article/supplementary material, further inquiries can be directed to the corresponding author.
